# Reducing Premature Mortality from Cardiovascular and Other Non-Communicable Diseases by One Third: Achieving Sustainable Development Goal Indicator 3.4.1

**DOI:** 10.5334/gh.531

**Published:** 2020-07-30

**Authors:** Thomas R. Frieden, Laura K. Cobb, Robynn C. Leidig, Sumi Mehta, Daniel Kass

**Affiliations:** 1 Resolve to Save Lives, New York, NY, US; 2 Vital Strategies, New York, NY, US

**Keywords:** noncommunicable disease prevention, cardiovascular disease prevention, public health interventions, public health policy, global health, tobacco control

## Abstract

Non-communicable diseases (NCDs) are the world’s leading causes of death and disability, with cardiovascular disease (CVD) accounting for half of NCD deaths. An ambitious global target established by the United Nations Sustainable Development Goals – indicator 3.4.1 – aims to reduce the risk of premature death among people aged 30–69 years from CVD, cancer, diabetes, and chronic lung disease by one third by 2030. This article reviews the science and practice informing what is required to achieve this target, identifying seven interventions that can accelerate progress: 1) tobacco control; 2) treatment to reduce cardiovascular risk; 3) reduction of dietary sodium; 4) reduction of household air pollution; 5) elimination of artificial trans fat; 6) reduction of alcohol use; and 7) prevention, detection, and treatment of cancers. Achieving the target is possible – there has already been progress in some areas, particularly related to CVD reduction – but only if there is faster, more concerted action.

## Introduction

Cardiovascular disease (CVD) and other non-communicable diseases (NCDs) are the world’s leading causes of death and disability, are the main driver of increasing health care costs, and undermine the economic stability of individuals, families, communities, and countries [1,2]. CVD kills more people than any other cause, but most CVD deaths are preventable with currently available interventions. Many opportunities to prevent disease, disability, and death are being missed, and, in the US and some other high-income countries, the decrease in CVD that has driven the increase in life expectancy has stalled or begun to reverse in recent years [3]. In some low- and middle-income countries, death rates from NCDs are twice those of high-income countries [4], and, with population growth and aging, the number of cases in these countries is increasing [5]. This article provides a focused overview of the highest impact public health approaches to the prevention of cardiovascular and other leading non-communicable diseases. Most progress reducing illness, disability, and death over the past century has come from public health interventions, ranging from clean water to tobacco control to reduced cholesterol [6,7]. A public health approach is, similarly, the most likely means to accelerate progress reducing CVD globally in the coming decades.

In 2011, the United Nations General Assembly and, subsequently, the United Nations Sustainable Development Goals (SDG) committed the world to addressing NCDs [8,9]. SDG indicator 3.4.1 is a one-third reduction in the risk of death from CVD, cancer, diabetes, and chronic lung disease among people aged 30–69 years between 2015 and 2030 [10]. Among these four targeted causes of death, cardiovascular disease is the leading cause, accounting for half of deaths in this age group. Although concerns have been raised that a mortality reduction target may under-value interventions that reduce disability, preventing avoidable death is a goal that is understandable, correlates with decreased disability, and is of paramount importance. This article examines what is required to achieve this target – which is also reflected in the World Health Organization (WHO) plan to achieve a 25% reduction in premature NCD-related deaths between 2010 and 2025 [11] and its updated workplan reflecting poor progress through 2015 [12] – focusing on specific steps that can be taken to track and accelerate global action.

Other analyses have looked at global trends in mortality [1,13] and the means to achieve other NCD targets [14], and we have earlier documented the ability of focused interventions to reduce deaths from cardiovascular disease [15]. The current article, which builds on the earlier WHO workplans and guidance, appears to be the first to quantify with specificity what is required to achieve SDG 3.4.1; the article also reviews the specific actions needed to achieve this goal. Although global NCD death rates are declining, having decreased at a rate of approximately 1.1% annually from 2010 to 2015, achieving SDG 3.4 would require doubling the current rate of decrease from 2015–2030. A pragmatic, transparent, and understandable way to approximate preventable deaths is to estimate the proportion of 2015 deaths that could be prevented, without considering trends or regional variation.

WHO has identified ‘Best Buy’ and other cost-effective interventions that can be implemented widely and cost-effectively [16]. Of the approximately 12.1 million deaths among people aged 30–69 years in 2015 from the four targeted NCDs (Table 1), approximately 6 million were from CVD, 4.5 million from cancer, 880,000 from diabetes, and 780,000 from chronic lung disease [17]. To determine whether and how the one-third reduction is possible, we analyzed interventions which met two criteria: those already implemented widely in at least some locations and which have been documented to reduce mortality from the targeted NCDs.

**Table 1:** Accelerator interventions which if implemented can enable the world to reach the target of United Nations Sustainable Development Goal Target 3.4.1 of a one-third reduction in death among people aged 30–69 years between 2015 and 2030.

Risk Factor/Disease	Target percent reduction to achieve SDG 3.4.1	Estimated reduction deaths from selected NCDs, ages 30–69 (see Web Annex)	Core indicator for accountability and global baseline	Means to achieve target

Tobacco use	50% prevalence reduction	13.0%	Prevalence of smoking: 20%	WHO MPOWER package,* [57] currently fully implemented for less than 0.5% of the world’s population, [58] and, potentially, new measures to reduce the addictiveness (nicotine delivery) of tobacco products
Excess sodium consumption	30% consumption reduction	5.7%	Sodium consumption: >95% of world population consumes more than WHO-recommended amount of sodium. [59]	WHO SHAKE technical package* [60]
Cervical,* liver, colon, and other cancers	27% mortality reduction overall (20–67%, depending on type of cancer; see Web Annex)	5.4%	HBV and HPV vaccination of the target population (currently 43% and 40% respectively) [61,62]. Detection, screening, and treatment of preventable or treatable cancers (not done for much of the world).	HBV and HPV* vaccination. Detection, screening, and treatment of cervical* and preventable or treatable cancers.
Hypertension	50% hypertension control	5.0%	Hypertension control – current rate 14% [34]	WHO HEARTS technical package* [63]
Artificial trans fat consumption	100% reduction	2.0%	Consumption of artificial trans fat. 8% of world population protected by best-practice policies to eliminate artificial trans fat [52,53].	WHO REPLACE action package [67]
Household air pollution	25% reduction in use of solid fuel for cooking	1.4%	Percent of population using solid fuel for cooking – currently 35% [64].	World Bank: Household Energy for Cooking Project Design Principles [65] WHO guidelines for indoor air quality: household fuel consumption [66]
Harmful alcohol use	20% reduction	0.6%	Per capita alcohol consumption – 15.1 liters pure alcohol equivalent annually per current adult drinker [41].	WHO SAFER technical package* [42]
Total		33.1%		

* WHO Best Buy.

## Intervention Areas to Accelerate Progress

Seven areas for intervention – accelerators – could result in substantial reductions in the targeted causes of death. These interventions are particularly important for the reduction of CVD. Since CVD is the leading cause of death globally and the leading cause of death among the four causes targeted by SDG 3.4.1, achievement of the global target will depend, to a great extent, on prevention and control of CVD. Different targets could be set for each indicator and still achieve the overall SDG one-third reduction goal, with more progress in one area offsetting less progress in another. However, it is feasible for the targets suggested here to be reached by the 2030 SDG end date provided that suggested interventions have the expected impact.

### Intervention Areas to Prevent and Control CVD

#### Tobacco control

Tobacco is the world’s leading preventable cause of death, with 7 million deaths each year caused by use of tobacco products and exposure to secondhand smoke. Approximately one-fourth of all CVD is caused by tobacco use [18]. Unless there is substantial progress reducing tobacco use, SDG 3.4.1 will not be reached; in the suggested approach, as in others determining what is required for rapid global progress [1], nearly half of all mortality reduction is driven by reduction in tobacco use. The WHO MPOWER package reduces tobacco use and related illness and death. Increasing taxes is the single most effective intervention to reduce tobacco consumption and encourage cessation. Tax revenues increase even as consumption declines, and taxes on these products ‘disproportionately benefit low-income households’ [19]. Creating smoke-free environments improves health and encourages cessation. Changing the image of tobacco by limiting or banning tobacco marketing as well as running hard-hitting counter-advertising campaigns and enforcing plain packaging and/or graphic cigarette pack warnings further reduces consumption. Even in countries, such as the United States, that have made progress reducing tobacco use, much more progress will be needed: tobacco use remains the leading preventable cause of death in the United States. To achieve the ambitious target of a 50% reduction in tobacco use, full implementation of MPOWER, particularly large and effective taxes on tobacco products, and, possibly, new approaches such as the mandatory reduction of nicotine in combustible tobacco will be needed.

#### Treatment to reduce cardiovascular risk, particularly by treating hypertension

High blood pressure kills more than 10 million people a year, more than any other condition and more than all infectious diseases combined [17]. Although there are well-established treatment regimens with inexpensive, well-tolerated, and readily available generic medications that have been standard-of-care in high-income countries for half a century, many people are not screened and many who are diagnosed are not on effective treatment, with the result that more than 85% of people in the world with hypertension, and approximately 50% of people in the United States, do not have it controlled to less than 140/90 mm Hg. Globally, several approaches have been proposed to reduce cardiovascular risk with medications. These include widespread use of polypills (including for primary prevention) [20,21,22], use of risk-assessment to identify and treat patients most likely to have a cardiovascular event in the coming years [23,24], and combinations of three low-dose anti-hypertensive medications with little or no dose titration [25,26,27]. Our initiative, Resolve to Save Lives, along with WHO, the American Heart Association, and more than nine other organizations, is partnering with countries to implement a blended approach that includes standardized drug- and dose-specific treatment protocols (ideally including two-drug fixed dose combinations of anti-hypertensive medications) [28], ensured drug supply, team-based care for adherence support and ideally dosage titration, patient-centered services including free care and ready access to blood pressure measurement, and rigorous real-time monitoring for continuous program improvement [29,30]. Whatever approach is used, a substantial increase in the proportion of people who would benefit from cardiovascular medications need to receive them, and whatever approach is needed will require improvements in primary care. Although this is a daunting challenge, other public health programs provide hope and a potential model [31]. Globally there is both commitment to and progress toward the ‘90-90-90’ goal for HIV: detecting 90% of those with HIV infection, starting 90% of those detected on treatment with anti-retroviral agents, and achieving treatment success (viral load suppression) in 90% of those started on treatment, which would result in an overall 73% rate of control [32]. Some countries are close to achieving this goal, and, globally, despite HIV treatment being substantially more medically complex and much more expensive than treatment of hypertension, current detection, treatment, and control rates for HIV are much higher than those for hypertension (81% vs. 52%, 67% vs. 35%, and 59% vs. 14%, respectively) [33,34]. Of all adult primary care interventions, improvement in management of hypertension treatment has the potential to save the most lives [35]. At least post-CVD event, aspirin and statins significantly improve outcomes, although the population-wide effect of this intervention will be smaller than the impact of widespread treatment of a high proportion of at-risk people. Use of statins in high-risk people pre-CVD event, although the subject of some disagreement, could also reduce cardiovascular disease, and could be done in the same programs that treat hypertension.

#### Sodium reduction

The vast majority of people consume far more sodium than necessary to meet physiological need, with average consumption in many countries more than twice the recommended daily intake [36,37]. Excess sodium increases blood pressure and the risk of cardiovascular disease [38,39,40]. Reducing sodium consumption is possible by ensuring that packaged and restaurant foods meet reasonable standards for steadily decreasing sodium levels. Product reformulation is feasible, and consumer tastes adapt to food with lower sodium content, especially if phased in over several years. In areas where most sodium is added during cooking at home, changing food preparation patterns will be necessary. Promotion of low-sodium, potassium-enriched salts (e.g., 15–25% potassium) could have an important role as well.

#### Reduction of harmful alcohol use

In 2016, an estimated 600,000 CVD deaths were caused by alcohol consumption, including 10% of fatal hemorrhagic strokes and nearly 3% of fatal cardiac ischemia [41]. As with tobacco, increasing taxes and prices reduces alcohol consumption and associated deaths. Limits on who can buy alcohol as well as how, when, and where it is sold and served are also effective interventions. Modifying the social environment around alcohol, including restrictions on advertising and marketing, can also reduce alcohol use [42]. In addition to reducing deaths from NCDs, reduction of harmful alcohol use would also greatly reduce fatal road traffic crashes, violence, tuberculosis, HIV, and sexually transmitted diseases, as well as many other health and social harms targeted by other Sustainable Development Goals [41].

#### Artificial trans fat elimination

Consumption of industrially produced artificial trans fat increases the risk of heart attack and cardiovascular death [43]. Consumers have little control over the content of foods they do not prepare themselves. Reformulation to use healthy alternatives will not increase the cost of food, make food taste different, or prevent any foods from being manufactured; therefore a complete ban on partially hydrogenated vegetable oils, or a maximum limit of 2% trans fat in all foods has been recommended by the World Health Organization and others [44].

#### Reduction of household air pollution

Use of solid fuels (e.g., wood, charcoal, dung) for cooking, heating, and lighting is common in many low- and low-middle-income countries; approximately 40% of air pollution-related deaths are caused by CVD [45,46]. Shifting to cleaner household energy sources (e.g., propane, electricity) will require expansion of the supply chains for affordable fuels as well as consumer education and changes in household equipment. Reduction in ambient air pollution, including through cleaner power generation and improved mass transportation, would also have large health benefits, but current trends are not favorable.

### Other Intervention Areas to Address the SDG Target

#### Prevention, detection, and treatment of cervical, liver, colon, and other cancers

Some types of cancer are largely preventable by vaccination, risk-factor modification, or early detection and care. Many cervical and liver cancers can be prevented by vaccination against human papilloma virus and hepatitis B virus, respectively [47]. Approximately one-third of deaths from cancer are due to the five leading behavioral and dietary risks: high body mass index, low fruit and vegetable intake, lack of physical activity, tobacco use, and alcohol use [48]. Tobacco use is the most important risk factor for cancer and is responsible for approximately 22% of cancer deaths [49]. Some of the most common cancer types, such as breast, cervical, oral, and colorectal cancers, have high cure rates when detected early and treated according to best practices; detecting colorectal cancers when still localized results in a 90% survival rate [50]. Cancer treatment has advanced greatly in recent years, but readily treatable cancers are often neither diagnosed nor treated in many countries.

## Amenability to Action

Although there is some overlap in the people affected by these numbers, achievement of the SDG target appears to be theoretically possible – but only if there is more rapid and concerted action, including for interventions which require substantial political and some financial capital. The SDG target will not be met unless there is an urgent change in approach, particularly given the commercial interests working against effective action on tobacco, alcohol, unhealthy food, healthy environments, and other determinants of disease. Scaling up programs that require high-functioning primary health care systems is challenging, particularly in countries with limited financing and shortages of trained health workers. Task sharing, also known as team-based care, in which non-physician health providers or lay community health workers assume a larger role in patient care, can improve health system functioning and improve patient outcomes.

Progress reducing CVD risk factors is not only theoretically possible – it has been achieved in some areas. In less than five years, five countries (Brazil, India, Philippines, Russia, and Ukraine) implemented parts of the WHO-recommended MPOWER strategy and decreased smoking by 11–22%, resulting in at least 30 million fewer smokers and preventing more than 10 million future deaths [51]. Dozens of countries have enacted regulations to eliminate artificial trans fat from the food supply [52]. Chile has enacted best-practice nutritional policies that can substantially reduce sodium intake [54]. Canada has demonstrated that it is possible to achieve hypertension control in more than two-thirds of all people with hypertension in the country – roughly five times the global control rate of 10–15% [55]. It took India less than four years to provide access to clean household energy to more than 80 million poor households [56].

These interventions can also help countries improve health more generally. Removing artificial trans fat strengthens country regulatory capacity critical for many nutrition and food safety interventions. Hypertension control is a pathfinder for primary health care. And reduction in household air pollution and in alcohol consumption will have many health and societal benefits. Advocacy will be important to achieving progress in many of these areas, particularly where there are strong industry interests that may resist change. Engagement of civil society, health professional societies, and media is especially important for strategies that seek to prevent disease through changes in policies that may not be in the interest of powerful commercial groups such as the tobacco industry.

One important means to increase the likelihood that the SDG 3.4.1 target will be met is to increase accountability. Although the impact of different interventions will differ by region, country, gender, and other factors, and some interventions are more or less important for some populations, these are the interventions that can achieve the global SDG target. Each can be assessed independently on an annual basis to determine the level of progress at country, regional, and global levels. Progress can be accelerated by applying a rigorous monitoring framework to ensure accurate, timely measurement of and increased attention to and accountability for the actions needed as well as the impact these measures have on risk factors and deaths.

## Limitations

The current analysis does not account for temporal trends, age patterns, or sex differences in deaths or in risk factors, differences among countries, regions, and income level, or for the risk of death versus the number of deaths, or for the time it would take for a change in risk factors to result in a change in mortality. Cardiovascular mortality decreases rapidly after smoking cessation, blood pressure control, and other interventions, and cancer treatment can increase survival promptly, whereas the decrease in cancer risk after tobacco cessation takes years. We did not include in this analysis the WHO Best Buy of community-wide education on physical activity; we could not identify a geographic area which has scaled this intervention widely and documented a population-wide effect.

Some interventions not included in this analysis may be useful even if there is no current evidence of efficacy. For example, taxation of sugary beverages is likely to reduce intake and help reduce the risk of obesity and its associated morbidity and mortality; only interventions shown to have been effectively scaled up and to have reduced the targeted cause of death are included in this analysis. (Taxes on sugary beverages have been documented to reduce consumption of sugary drinks, but not yet to have reduced obesity, diabetes, or related mortality.) We also did not model glycemic control for people with diabetes, as we could not determine the mortality benefit of this intervention. Finally, there may be overlap among the deaths averted, although there are also synergies among interventions that are not accounted for.

We limited our analysis to the 2015 baseline year as this was the starting point of the 15-year SDG target. There has since been limited to no progress in most of the SDG 3.4.1 indicator areas, and none are currently on track to meet the targets established for 2030. As a result, we believe that this analysis and the suggested interventions remain fundamentally valid; assessment of interim progress could be the subject of additional research and analysis.

## Conclusion

Although the world is not currently on track to reach its goals in reducing premature death from NCDs, there has been some progress since implementation of the WHO global action plan, and it is possible to achieve the SDG 3.4.1 target. Progress requires courageous political decisions (e.g., to tax and limit marketing and promotion of tobacco and alcohol, and to authorize nurses and other allied health workers to provide hypertension and cancer care according to physician-determined protocols) as well as increased investment in prevention and primary care throughout the health system. Cardiovascular and cancer control will require not just access and financial protection, which is the current global focus for universal health coverage, but also accountability for specific, high-impact health outcomes, especially hypertension and other CVD management, vaccination, and cancer detection and management.

The pace of implementation is critically important. Every year of delay will make the task more difficult, cause millions of people to become disabled or die from preventable conditions, and cost billions of dollars in avoidable health care spending. By holding governments accountable for implementation of key accelerators of progress such as the seven outlined here, and if the medical community advocates more strongly for investment of the resources and accountability needed to achieve results, the world can reach the target.

## Additional File

The additional file for this article can be found as follows:

10.5334/gh.531.s1 Supplementary File. Web Annex.

## References

[1] NCD Countdown 2030 collaborators. NCD Countdown 2030: Worldwide trends in non-communicable disease mortality and progress towards Sustainable Development Goal target 3.4. Lancet. 2018 9 22; 392(10152): 1072–1088. DOI: 10.1016/S0140-6736(18)31992-5 30264707

[2] Council on Foreign Relations. The emerging global health crisis: Noncommunicable diseases in low- and middle-income countries New York: Council on Foreign Relations; 2014 https://www.cfr.org/report/emerging-global-health-crisis (accessed 11 June 2020).

[3] Kochanek KD, Murphy SL, Xu J, Arias E. Deaths: Final data for 2017 National Vital Statistics Reports. 2019; 68(9). Hyattsville, MD: National Center for Health Statistics https://www.cdc.gov/nchs/data/nvsr/nvsr68/nvsr68_09-508.pdf (accessed 11 June 2020). 32501199

[4] World Health Organization. World health statistics 2019: Monitoring health for the SDGs Geneva: World Health Organization; 2019 https://www.who.int/gho/publications/world_health_statistics/2019/en (accessed 11 June 2020).

[5] Department of Economic and Social Affairs. Population facts: Population ageing and the non-communicable diseases New York: Department of Economic and Social Affairs; 2012 https://www.un.org/en/development/desa/population/publications/pdf/popfacts/popfacts_2012-1.1.pdf (accessed 11 June 2020).

[6] Centers for Disease Control and Prevention (CDC). Ten great public health achievements – United States, 1900–1999. MMWR Morb Mortal Wkly Rep. 1999 4 2; 48(12): 241–243. 10220250

[7] Carroll MD, Fryar CD, Duong T, Nguyen DT. Total and high-density lipoprotein cholesterol in adults: United States, 2015–2016 NCHS Data Brief No. 290. Hyattsville, MD: National Center for Health Statistics; 2017 https://www.cdc.gov/nchs/data/databriefs/db290.pdf (accessed 11 June 2020).

[8] Political declaration of the high-level meeting of the General Assembly on the prevention and control of non-communicable diseases New York: United Nations; 2012 http://www.who.int/nmh/events/un_ncd_summit2011/political_declaration_en.pdf (accessed 11 June 2020).

[9] Sustainable Development Goals. Goal 3: Ensure healthy lives and promote well-being for all at all ages New York: United Nations; 2015 https://www.un.org/sustainabledevelopment/health (accessed 11 June 2020).

[10] Indicators and a monitoring framework: Launching a data revolution for the Sustainable Development Goals New York: Sustainable Development Solutions Network; 2015 http://indicators.report/targets/3-4 (accessed 11 June 2020).

[11] World Health Organization. Global action plan for the prevention and control of noncommunicable diseases 2013–2020 Geneva: World Health Organization; 2013 https://www.who.int/nmh/events/ncd_action_plan/en (accessed 11 June 2020).

[12] Governance: Updating Appendix 3 of the WHO global NCD action plan 2013–2020. Preparation for the third High-level Meeting of the General Assembly on the Prevention and Control of Non-communicable Diseases, to be held in 2018 Geneva: World Health Organization; 2016 https://www.who.int/ncds/governance/appendix3-update/en (accessed 11 June 2020).

[13] Norheim OF, Jha P, Admasu K, et al. Avoiding 40% of the premature deaths in each country, 2010–30: Review of national mortality trends to help quantify the UN sustainable development goal for health. Lancet. 2015 1 17; 385(9964): 239–252. DOI: 10.1016/S0140-6736(14)61591-9 25242039

[14] Kontis V, Mathers CD, Rehm J, et al. Contribution of six risk factors to achieving the 25×25 non-communicable disease mortality reduction target: A modelling study. Lancet. 2014 8 2; 384(9941): 427–437. DOI: 10.1016/S0140-6736(14)60616-4 24797573

[15] Kontis V, Cobb LK, Mathers CD, Frieden TR, Ezzati M, Danaei G. Three public health interventions could save 94 million lives in 25 years. Circulation. 2019 8 27; 140(9): 715–725. DOI: 10.1161/CIRCULATIONAHA.118.038160 31177824 PMC6727958

[16] Tackling NCDs. “Best buys” and other recommended interventions for the prevention and control of noncommunicable diseases Geneva: World Health Organization; 2017 https://apps.who.int/iris/handle/10665/259232 (accessed 11 June 2020).

[17] Global health estimates. 2016: Disease burden and mortality estimates: Cause-specific mortality, 2000–2016 Geneva: World Health Organization; 2018 https://www.who.int/healthinfo/global_burden_disease/estimates/en (accessed 11 June 2020).

[18] The health consequences of smoking – 50 years of progress. A report of the Surgeon General Atlanta: U.S. Department of Health and Human Services, Centers for Disease Control and Prevention, National Center for Chronic Disease Prevention and Health Promotion, Office on Smoking and Health; 2014 https://www.cdc.gov/tobacco/data_statistics/sgr/50th-anniversary/index.htm (accessed 11 June 2020).

[19] Furman J. Six lessons from the U.S. experience with tobacco taxes. In: Proceedings of Winning the Tax Wars: Global Solutions for Developing Countries, 24 May 2016 Washington: World Bank Conference https://obamawhitehouse.archives.gov/sites/default/files/page/files/20160524_cea_tobacco_tax_speech.pdf (accessed 11 June 2020).

[20] Roshandel G, Khoshnia M, Poustchi H, et al. Effectiveness of polypill for primary and secondary prevention of cardiovascular diseases (PolyIran): a pragmatic, cluster-randomised trial. Lancet. 2019 8 24; 394(10199): 672–683. DOI: 10.1016/S0140-6736(19)31791-X 31448738

[21] Selak V, Webster R, Stepien S, et al. Reaching cardiovascular prevention guideline targets with a polypill-based approach: a meta-analysis of randomised clinical trials. Heart. 2019 1; 105(1): 42–48. DOI: 10.1136/heartjnl-2018-313108 29954855

[22] Muñoz D, Uzoije P, Reynolds C, et al. Polypill for cardiovascular disease prevention in an underserved population. N Engl J Med. 2019 9 19; 381(12): 1114–1123. DOI: 10.1056/NEJMoa1815359 31532959 PMC6938029

[23] Prevention of cardiovascular disease: Guidelines for assessment and management of cardiovascular risk Geneva: World Health Organization; 2007 https://www.who.int/cardiovascular_diseases/guidelines/Full%20text.pdf (accessed 11 June 2020).

[24] Lloyd-Jones DM, Braun LT, Ndumele CE, et al. Use of risk assessment tools to guide decision-making in the primary prevention of atherosclerotic cardiovascular disease: A special report From the American Heart Association and American College of Cardiology. Circulation. 2019 6 18; 139(25): e1162–e1177. DOI: 10.1161/CIR.0000000000000638 30586766

[25] Webster R, Salam A, de Silva HA, et al. TRIUMPH Study Group. Fixed low-dose triple combination antihypertensive medication vs usual care for blood pressure control in patients with mild to moderate hypertension in Sri Lanka: a randomized clinical trial. JAMA. 2018 8 14; 320(6): 566–579. DOI: 10.1001/jama.2018.10359 30120478 PMC6583010

[26] Düsing R, Waeber B, Destro M, Santos Maia C, Brunel P. Triple-combination therapy in the treatment of hypertension: a review of the evidence. J Hum Hypertens. 2017 8; 31(8): 501–510. DOI: 10.1038/jhh.2017.5 28230062

[27] Salam A, Atkins ER, Hsu B, Webster R, Patel A, Rodgers A. Efficacy and safety of triple versus dual combination blood pressure-lowering drug therapy: a systematic review and meta-analysis of randomized controlled trials. J Hypertens. 2019 8; 37(8): 1567–1573. DOI: 10.1097/HJH.0000000000002089 31058799

[28] Kishore SP, Salam A, Rodgers A, Jaffe MG, Frieden T. Fixed-dose combinations for hypertension. Lancet. 2018 9 8; 392(10150): 819–820. DOI: 10.1016/S0140-6736(18)31814-2 30215377

[29] Frieden TR, Bloomberg MR. Saving an additional 100 million lives. Lancet. 2018 2 17; 391(10121): 709–712. DOI: 10.1016/S0140-6736(17)32443-1 28916368

[30] Frieden TR, Varghese CV, Kishore SP, et al. Scaling up effective treatment of hypertension – a pathfinder for universal health coverage. J Clin Hypertens (Greenwich). 2019 10; 21(10): 1442–1449. DOI: 10.1111/jch.13655 31544349 PMC8030517

[31] Angell SY, De Cock KM, Frieden TR. A public health approach to global management of hypertension. Lancet. 2015 2 28; 385(9970): 825–827. DOI: 10.1016/S0140-6736(14)62256-X 25752181 PMC4830267

[32] 90-90-90: An ambitious treatment target to help end the AIDS epidemic Geneva: Joint United Nations Programme on HIV/AIDS (UNAIDS); 2019 https://www.unaids.org/sites/default/files/media_asset/90-90-90_en.pdf (accessed 11 June 2020).

[33] Global AIDS update 2020. Seizing the moment: Tackling entrenched inequalities to end epidemics Geneva: Joint United Nations Programme on HIV/AIDS (UNAIDS); 2020 https://www.unaids.org/sites/default/files/media_asset/2020_global-aids-report_en.pdf (accessed 20 July 2020).

[34] Mills KT, Bundy JD, Kelly TN, et al. Global disparities of hypertension prevalence and control: A systematic analysis of population-based studies from 90 countries. Circulation. 2016 8 9; 134(6): 441–450. DOI: 10.1161/CIRCULATIONAHA.115.018912 27502908 PMC4979614

[35] Farley TA, Dalal MA, Mostashari F, Frieden TR. Deaths preventable in the U.S. by improvements in use of clinical preventive services. Am J Prev Med. 2010 6; 38(6): 600–609. DOI: 10.1016/j.amepre.2010.02.016 20494236

[36] Mozaffarian D, Fahimi S, Singh GM, et al. Global Burden of Diseases Nutrition and Chronic Diseases Expert Group. Global sodium consumption and death from cardiovascular causes. N Engl J Med. 2014 8 14; 371(7): 624–634. DOI: 10.1056/NEJMoa1304127 25119608

[37] GBD 2017 Diet Collaborators. Health effects of dietary risks in 195 countries, 1990–2017: a systematic analysis for the Global Burden of Disease Study 2017. Lancet. 2019 5 11; 393(10184): 1958–1972. DOI: 10.1016/S0140-6736(19)30041-8 30954305 PMC6899507

[38] Cappuccio FP, Beer M, Strazzullo P. European Salt Action Network. Population dietary salt reduction and the risk of cardiovascular disease. A scientific statement from the European Salt Action Network. Nutr Metab Cardiovasc Dis. 2018 12 7; 29(2): 107–114. DOI: 10.1016/j.numecd.2018.11.010 30583888

[39] Campbell NRC, He FJ, Tan M, et al. The International Consortium for Quality Research on Dietary Sodium/Salt (TRUE) position statement on the use of 24-hour, spot, and short duration (<24 hours) timed urine collections to assess dietary sodium intake. J Clin Hypertens (Greenwich). 2019 6; 21(6): 700–709. DOI: 10.1111/jch.13551 PMC6874851 31087778

[40] He FJ, Campbell NRC, Ma Y, MacGregor GA, Cogswell ME, Cook NR. Errors in estimating usual sodium intake by the Kawasaki formula alter its relationship with mortality: implications for public health. Int J Epidemiol. 2018 12 1; 47(6): 1784–1795. DOI: 10.1093/ije/dyy114 30517688 PMC6280933

[41] World Health Organization. Global status report on alcohol and health 2018 Geneva: World Health Organization; 2018 https://www.who.int/substance_abuse/publications/global_alcohol_report/gsr_2018/en (accessed 11 June 2020).

[42] SAFER: Preventing and reducing alcohol-related harms Geneva: World Health Organization; 2018 https://www.who.int/substance_abuse/safer/msb_safer_framework.pdf (accessed 11 June 2020).

[43] Wang Q, Afshin A, Yakoob MY, et al. Global Burden of Diseases Nutrition and Chronic Diseases Expert Group (NutrtiCoDE). Impact of nonoptimal intakes of saturated, polyunsaturated, and trans fat on global burdens of coronary heart disease. J Am Heart Assoc. 2016 1 20; 5(1). DOI: 10.1161/JAHA.115.002891 PMC4859401 26790695

[44] World Health Organization. Countdown to 2023: WHO report on global trans fat elimination 2019 Geneva: World Health Organization; 2019 https://apps.who.int/iris/bitstream/handle/10665/331300/9789241516440-eng.pdf (accessed 11 June 2020).

[45] Rajagopalan S, Al-Kindi SG, Brook RD. Air pollution and cardiovascular disease: JACC state-of-the-art review. J Am Coll Cardiol. 2018 10 23; 72(17): 2054–2070. DOI: 10.1016/j.jacc.2018.07.099 30336830

[46] Brook RD, Rajagopalan S, Pope, CA 3rd, et al. American Heart Association Council on Epidemiology and Prevention, Council on the Kidney in Cardiovascular Disease, and Council on Nutrition, Physical Activity and Metabolism. Particulate matter air pollution and cardiovascular disease: An update to the scientific statement from the American Heart Association. Circulation. 2010 6 1; 121(21): 2331–2378. DOI: 10.1161/CIR.0b013e3181dbece1 20458016

[47] Vaccines In: Jemal A, Torre L, Soerjomataram I, Brat, F (eds.), The cancer atlas. 2019; 29–30. 3rd ed. Atlanta: American Cancer Society.

[48] World Health Organization. Cancer Geneva: World Health Organization; 2019 http://www.who.int/cancer/en (accessed 11 June 2020).

[49] GBD 2015 Risk Factors Collaborators. Global, regional, and national comparative risk assessment of 79 behavioural, environmental and occupational, and metabolic risks or clusters of risks, 1990-2015: A systematic analysis for the Global Burden of Disease Study 2015. Lancet. 2016 10; 388(10053): 1659–1724. DOI: 10.1016/S0140-6736(16)32632-0 27733284 PMC5388856

[50] Survival Rates for Colorectal Cancer Atlanta: American Cancer Society; 2018 https://www.cancer.org/cancer/colon-rectal-cancer/detection-diagnosis-staging/survival-rates.html (accessed 11 June 2020).

[51] Data from: Tobacco Free Institute (TFI): Global Adult Tobacco Survey (GATS), individual country reports Geneva: World Health Organization; 2018 http://www.who.int/tobacco/surveillance/survey/gats/en (accessed 11 June 2020).

[52] Global database on the Implementation of Nutrition Action (GINA): TFA country score card Geneva: World Health Organization; 2012 https://extranet.who.int/nutrition/gina/en/scorecard/TFA (accessed 11 June 2020).

[53] World population prospects 2019 New York: United Nations Department iof Economic and Social Affairs 2019 https://population.un.org/wpp/Download/Standard/Population (accessed 20 July 2020).

[54] Corvalán C, Reyes M, Garmendia ML, Uauy R. Structural responses to the obesity and non-communicable diseases epidemic: Update on the Chilean law of food labelling and advertising. Obes Rev. 2019 3; 20(3): 364–374. DOI: 10.1111/obr.12802 30549191

[55] Padwal RS, Bienek A, McAlister FA, Campbell NR. Outcomes Research Task Force of the Canadian Hypertension Education Program. Epidemiology of hypertension in Canada: an update. Can J Cardiol. 2016 5; 32(5): 687–694. DOI: 10.1016/j.cjca.2015.07.734 26711315

[56] Tripathi A, Sagar A. Ujjwala 2.0: What should be done next? In: Harish S, Smith KR, eds. Ujjwala 2.0: From access to sustained usage. New Delhi: Collaborative Clean Air Policy Centre, New Delhi; 2019 https://static1.squarespace.com/static/53856e1ee4b00c6f1fc1f602/t/5d77fa14d48c3c428580b5fc/1568143894592/CCAPC-Ujjwala+V2.0-Aug+2019+%28002%29.pdf (accessed 11 June 2020).

[57] World Health Organization. MPOWER: A policy package to reverse the tobacco epidemic Geneva: World Health Organization; 2008 http://www.who.int/tobacco/mpower/mpower_english.pdf (accessed 11 June 2020).

[58] World Health Organization. WHO report on the global tobacco epidemic, 2019: Offer help to quit tobacco use Geneva: World Health Organization; 2019 https://www.who.int/tobacco/global_report/en (accessed 11 June 2020).

[59] Powles J, Fahimi S, Micha R, et al. Global Burden of Diseases Nutrition and Chronic Diseases Expert Group (NutriCoDE). Global, regional and national sodium intakes in 1990 and 2010: a systematic analysis of 24 h urinary sodium excretion and dietary surveys worldwide. BMJ Open. 2013 12 23; 3(12): e003733 DOI: 10.1136/bmjopen-2013-003733 (accessed 11 June 2020). PMC3884590 24366578

[60] World Health Organization. SHAKE the salt habit: The SHAKE technical package for salt reduction Geneva: World Health Organization; 2016 http://www.who.int/dietphysicalactivity/publications/shake-salt-habit/en (accessed 11 June 2020).

[61] World Health Organization. Immunization coverage: Key facts Geneva: World Health Organization, 2019 http://www.who.int/news-room/fact-sheets/detail/immunization-coverage (accessed 11 June 2020).

[62] Bruni L, Diaz M, Barrionuevo-Rosas L, et al. Global estimates of human papillomavirus vaccination coverage by region and income level: a pooled analysis. Lancet Glob Health. 2016 7; 4(7): e453–63. DOI: 10.1016/S2214-109X(16)30099-7 27340003

[63] World Health Organization. HEARTS: Technical package for cardiovascular disease management in primary health care Geneva: World Health Organization; 2016 http://www.who.int/cardiovascular_diseases/hearts/en (accessed 11 June 2020).

[64] State of global air. 2019 Boston: Health Effects Institute; 2019. https://www.stateofglobalair.org/data/#/air/plot (accessed 11 June 2020).

[65] Household energy for cooking: Project design principles Washington: The World Bank; 2013 http://documents.worldbank.org/curated/en/320081468183548304/Household-Energy-for-Cooking-Project-design-principles (accessed 11 June 2020).

[66] World Health Organization. WHO Guidelines for indoor air quality: household fuel combustion Geneva: World Health Organization; 2014 http://www.who.int/airpollution/guidelines/household-fuel-combustion/en (accessed 11 June 2020).

[67] World Health Organization. REPLACE trans fat: An action package to eliminate industrially-produced trans fat from the global food supply Geneva: World Health Organization; 2018 http://www.who.int/nutrition/topics/replace-transfat (accessed 11 June 2020).

