# Supplementary material for: Reducing Premature Mortality from Cardiovascular and Other Non-Communicable Diseases by One Third: Achieving Sustainable Development Goal Indicator 3.4.1
Source: Glob Heart. 2020 Jul 30;15(1):50. doi: 10.5334/gh.531 (PMC7427687; doi:10.5334/gh.531)
Supplement: Supplementary File. — Web Annex. [file gh-15-1-531-s1.pdf]

Web Annex

According to WHO estimates of mortality for 2015,[1] there were 12.1 million deaths among people age 30-69 from the four targeted causes, of the 30.9 million deaths from these causes among all age groups, as per the table below.

Cause	All ages	30-69 years old	Proportion 30-69
Malignant neoplasms	8.74 million	4.44 million	51%
Cardiovascular diseases (not including rheumatic heart disease)	17.21 million	5.96 million	35%
Diabetes mellitus (including chronic kidney disease from diabetes)	1.99 million	0.880 million	44%
Chronic Obstructive Pulmonary Disease	2.97 million	0.777 million	26%
**Total**	**30.9 million**	**12.1 million**	**39%**

Data on the risk attribution for these deaths is not available from the World Health Organization, except for household air pollution. For household air pollution,[2] the number of deaths for each of the four causes was multiplied by the proportion of all deaths from that cause in the 30-69-year-old age group to establish the estimated number of 1.16 million deaths from this cause in this age group.

Tobacco control — reduction of tobacco use by 50%
The WHO voluntary global NCD target for 2025 is a 30% reduction in tobacco use. We propose a more ambitious goal of a 50% reduction of tobacco use by 2030 in order to meet SDG 3.4.[3]

- In 2015, 4.5 million people between the ages of 30-69 years died from cancer, and 27% of these deaths were due to tobacco use (1.2 million).[4] Cessation of tobacco use would prevent 90% of these cancer deaths among quitters, resulting in **547,000 cancer deaths prevented** from a 50% reduction in tobacco use.[5]
- In 2015, 6 million people between the ages of 30-69 years died from cardiovascular disease; and 29% of these deaths were due to smoking (1.7 million).[4] A 50% reduction in tobacco use will delay about 95% of deaths from cardiovascular disease in this population,[5] resulting in **827,000 cardiovascular disease deaths prevented.**
- In 2015, 780,000 people aged 30-69 years died from chronic respiratory disease; and 45% of these deaths were due to smoking (351,000).[4] A 50% reduction in tobacco use will delay about 90% of deaths,[5] resulting in **158,000 chronic respiratory disease deaths prevented.**
- Total: 547,000 (cancer) + 827,000 (CVD) + 158,000 (chronic respiratory disease) = **1,532,000 deaths prevented from a 50% reduction of tobacco use**, representing 13% (1.532 million/12.1 million) of annual deaths due to NCDs covered in SDG 3.4.1.

Treatment of hypertension, sodium reduction, and artificial trans fat elimination:
Estimates for hypertension, sodium and trans fat are based on a modeling analysis by Kontis et al.[6] Baseline global and regional estimates of sodium intake, trans fat intake and hypertension treatment coverage were developed based on pooled population health surveys and observational studies. A

meta-analysis of epidemiological studies was used to derive relative risks and population-attributable fraction was used to estimate the proportional effect of each intervention on mortality rates. The paper estimated the number of deaths that would be delayed between 2015 and 2040 by increasing hypertension treatment coverage to 70%, reducing sodium intake by 30% and reducing mean population intake of trans fat to 0.5% of energy; 70% hypertension treatment is a proxy for 50% hypertension control, as there was insufficient evidence to model the impact of hypertension control. Similarly, TFA intake of 0.5% is a proxy for elimination of artificial trans fat. The numbers in Table 1 are unpublished estimates of the impact of these three interventions using 2015 population data for adults aged 30-69 years (Kontis, personal communication), and reflect numbers of deaths averted at 2015 rates of 245,300, 692,900, and 604,600, respectively for trans fat elimination, sodium reduction, and hypertension treatment. Although these numbers were not included in the published analysis, they were generated as part of that analysis using the published methodology.

Reduction of household air pollution (from solid fuel and/or kerosene) — 25% reduction in population exposed to household air pollution (HAP), and in each of the 25% of the total households with reduced exposure, there is only a 50% reduction in exposure to household-generated fine particulate matter.

- The proportion of households in a country relying mainly on polluting fuels and technologies for cooking is used as a proxy indicator to estimate population exposure to household air pollution. Currently, households using mainly coal, wood, charcoal, dung, crop residues and kerosene are considered exposed to HAP.[7]
- In 2015, 35% of the world was exposed to HAP.[7]
- More than 3 million people die prematurely each year from exposure to HAP, with an estimated 1.16 million of these being from the 4 causes targeted by SDG 3.8.1 in the 30-69 year age group.[2]
- We estimated adult deaths averted using the HAPIT tool,[8] using the following assumptions:
    - 25% reduction in the prevalence of exposure to HAP, leading to a 50% reduction in these households' exposure to particulate matter associated with HAP. This is a conservative estimate that takes into account the experience thus far with partial/incomplete transitions to cleaner fuels resulting in continued exposure to particulates in these households. The availability of clean fuels, principally liquified propane gas, typically reduces but does not eliminate HAP, as some households continue to use some solid fuels and/or are exposed to HAP from its continued use by nearby households that have not converted to clean fuels.
- Deaths averted are among adults from chronic respiratory disease, heart disease and stroke, and cancer of the trachea, bronchus, and lung.
- Country-specific impacts in India, Indonesia and Ghana were modeled to reflect a range of background (ambient) exposure levels to particulates and underlying disease distributions against which the HAP exposure reduction was evaluated.[9,10] From these estimates, we extrapolated to global HAP-exposed population based on the proportion of the global exposed population each country represented.
- We discounted the modeled impacts by 64% to account for the proportion of the burden of air pollution-related mortality in the most heavily affected countries that are not in the 30-69-year age group.

  A 25% reduction in the population exposed to HAP would prevent 168,000 annual deaths from these causes (1.4% of targeted deaths).

Reduction of harmful alcohol use — 20% reduction in mortality
There are approximately 3 million total alcohol deaths across all ages.[11] We estimate that 30-69-year-olds make up about 40% of the total deaths, which is (3 million*0.4) = 1.2 million deaths.

- The WHO Global monitoring framework for NCDs calls for a 10% reduction in the harmful use of alcohol.[12] Implementing the multiple evidence-based interventions in the SAFER technical package can have a substantial impact on consumption and mortality.[13] Across the WHO EURO region, alcohol consumption per capita declined 20% between 2005-2016 and heavy episodic drinking decreased 26%,[11] showing that a more ambitious target is possible.[14,15] Certain countries achieved even greater improvements: Russia recently experienced a 43% decline in per capita consumption, with an even larger reduction in heavy episodic drinking among males. These trends coincided with an approximately 50% reduction in cardiovascular disease mortality for males and females (although not all was attributable to alcohol policy).[16] In order to achieve SDG 3.4, we propose a 20% reduction in mortality from harmful alcohol use.
- Among the 1.2 million 30-69-year-olds who die from alcohol, about 12.5% are from cancer[4] (1.2 million*0.125) = 150,000. A reduction of 20% in alcohol-associated mortality would delay (150,000*0.20) = **30,000 cancer deaths**.
- Additionally, about 20% of alcohol-attributable deaths are from cardiovascular diseases[11] (1.2 million*0.20) = 240,000. Reduction of 20% would delay (240,000*0.20) = **48,000 cardiovascular disease deaths.**
- In total, reduction of alcohol-associated mortality by 20% would lead to (30,000 + 48,000) = **78,000 deaths prevented**, which prevents about 0.6% (78,000/12.1 million) of annual deaths due to SDG 3.4. The alcohol analysis for this purpose is restricted only to the four non-communicable disease outcomes and target age group as outlined in SDG 3.4. The health benefit of reducing harmful alcohol use is much higher when considering other health conditions, societal impacts, and disability, in addition to mortality.

Prevention, detection, and treatment of cancers — 27% reduction in mortality, beyond the reduction from decreases in risk factors of tobacco use, alcohol use, and household air pollution.

- **Cervical cancer**: In 2015, 206,000 women between the ages of 30-69 years died from cervical cancer.[1] Current HPV vaccination rate among the targeted population is about 40%.[17] However, considering that the vaccine does not cover all in this age group, many are already infected, coverage is imperfect, and follow-up rates after screening may be low, preventing 50% of deaths per year through screening and vaccination would result in **103,000 deaths prevented.**
- **Liver cancer**: In 2015, 466,000 people between the ages of 30-69 years died from liver cancer.[1] Through HBV vaccination (shown to decrease chronic HBV infection by 9-15% in the general population below 20 years of age[18]), and treatment of hepatitis B and C, an estimated 20% of deaths could be prevented, resulting in **93,000 deaths prevented.**
- **Colon cancer**: In 2015, 330,000 people between the ages of 30-69 years died from colon and rectal cancers.[1] If, through aggressive efforts, 25% of deaths per year are prevented, [19,20] that would result in **83,000 deaths prevented**.
- **Leukemia and lymphoma**: In 2015, 283,000 people between the ages of 30-69 years died from leukemia or lymphoma.[1] According to the CONCORD study, the 5-year survival rate for leukemia ranges from 30-50% in most countries and 40-70% for lymphoma,[21] with most such cancers

occurring in parts of the world where there is little or no treatment. If an additional 40% of deaths in this age group can be prevented through treatment, this will result in **113,000 deaths prevented**.

- **Breast cancer**: In 2015, 378,000 people between the ages of 30-69 years died from breast cancer.[1] Population-based mammography screening programs have been shown to reduce breast cancer mortality by around 20%, although less in low resource settings.[22] Combining screening and treatment, if 20% of breast cancer deaths in this age group are delayed through screening and treatment, that would result in **76,000 deaths prevented.**
- **Esophageal cancer**: In 2015, 224,000 people between the ages of 30-69 years died from esophageal cancer.[1] The effectiveness of treatment can vary from 20-90%, depending on stage and type of treatment.[23] If 20% of deaths could be delayed globally through treatment, that would result in **45,000 deaths prevented**.
- **Lip and oral cancer:** In 2015, 108,000 people between the ages of 30-69 years died from lip and oral cancer.[1] Lip and oral cancers can be treated by surgery and/or radiation therapy. The cure rate varies from 65-100%, depending on stage and location of cancer.[24] With an 82.5% cure rate and assuming that an additional 50% of lip and oral cancers could be treated globally, this would result in **45,000 deaths prevented.**
- **Bladder cancer:** In 2015, 58,000 people between the ages of 30-69 years died from bladder cancer.[1] Bladder cancer can be treated with surgery, radiation, biological agents, and chemotherapy. Studies have shown that the overall recurrence-free 5-year survival with this treatment is 64-68%.[25,26] A 66% cure rate and assuming that an additional 50% of bladder cancers could be treated globally would result in **19,000 deaths prevented.**
- **Non-melanoma skin cancer:** In 2015, 18,000 people between the ages of 30-69 years died from non-melanoma skin cancer.[1] Non-melanoma skin cancer is very common, and many effective treatments are available. If found and treated early, the 5-year relative survival rate is 95-100%.[27] If non-melanoma skin cancer deaths are reduced by half globally, this would result in **9,000 deaths prevented.**
- **Testicular cancer**: In 2015, 6,000 people between the ages of 30-69 years died from testicular cancer.[1] If detected and treated early, people with testicular cancer who receive adjuvant chemotherapy or radiation therapy have a cure rate of 90-100%.[28] Reducing testicular cancer deaths by half globally would result in **3,000 deaths prevented.**
- **Other malignant neoplasms**: In 2015, 298,000 people between the ages of 30-69 years died from other malignant neoplasms.[1] In general, 30-50% of all cancer deaths can be prevented.[29] Preventing 20% of deaths from other malignant neoplasms would result in an additional **60,000 deaths prevented.**
- Total deaths prevented from screening, vaccination, detection, and/or treatment of cancers: **648,000**, amounting to **5.4%** (648,000/12.1 million) of annual deaths targeted by SDG 3.4.

Type of cancer	Total deaths, 2015, (30-69)	Target percent reduction to achieve SDG 3.4	Estimated number of deaths prevented at 2015 rates
Cervical Cancer	206,000	0.50	103,000
Liver cancer	466,000	0.20	93,000
Colon cancer	330,000	0.25	83,000
Leukemia + Lymphoma	283,000	0.40	113,000
Breast cancer	378,000	0.20	76,000
Esophageal cancer	224,000	0.20	45,000
Lip and oral cancer	108,000	0.50	45,000
Bladder cancer	58,000	0.50	19,000
Non-melanoma skin cancer	18,000	0.50	9,000
Testicular cancer	6,000	0.50	3,000
Other malignant neoplasms	298,000	0.20	60,000
**TOTAL**	**2,377,000**	**~0.27**	**648,000**

References

[1] Global health estimates 2016: Disease burden and mortality estimates: Disease burden, 2000-2016. Geneva, World Health Organization; 2018 (https://www.who.int/healthinfo/global_burden_disease/estimates/en, accessed 11 June 2020).
[2] Global Health Observatory data repository. Household air pollution attributable deaths. Geneva, World Health Organization, 2016 (http://apps.who.int/gho/data/node.main.BODHOUSEHOLDAIRDTHS, accessed 11 June 2020).
[3] NCD global monitoring framework. Geneva, World Health Organization, 2013 (http://www.who.int/nmh/global_monitoring_framework/en, accessed 11 June 2020).
[4] Calculated from: Global Health Data Exchange. GBD Results Tool. Seattle: Institute for Health Metrics and Evaluation, 2018 (http://ghdx.healthdata.org/gbd-results-tool, accessed 11 June 2020).
[5] Crofton J, Simpson D. Tobacco: a global threat. Hong Kong, Macmillan Education, 2002.
[6] Kontis V, Cobb LK, Mathers CD, Frieden TR, Ezzati M, Danaei G. Three public health interventions could save 94 million lives in 25 years. Circulation. 2019 Aug 27;140(9):715-725. DOI: https://doi.org/10.1161/CIRCULATIONAHA.118.038160.
[7] Burden of disease from household air pollution for 2016: Description of method. Version 4. Geneva, World Health Organization, 2018 (http://www.who.int/airpollution/data/HAP_BoD_methods_May2018.pdf, accessed 11 June 2020).
[8] Pillarisetti A, Mehta, S; Smith KR. HAPIT, the Household Air Pollution Intervention Tool, to evaluate the health benefits and cost-effectiveness of clean cooking interventions. In: Thomas EA, ed., Broken pumps and promises: Incentivizing impact in environmental health. Heidelberg, Springer International Press, 2016: 147-169.
[9] Shupler M, Godwin W, Frostad J, Gustafson P, Arku RE, Brauer M. Global estimation of exposure to fine particulate matter ($PM_{2.5}$) from household air pollution. Environ Int. 2018 Nov;120:354-363. DOI: https://doi.org/10.1016/j.envint.2018.08.026.
[10] State of global air 2019. Boston: Health Effects Institute, 2019 (https://www.stateofglobalair.org, accessed 11 June 2020).
[11] Global status report on alcohol and health 2018. Geneva: World Health Organization, 2018 (https://www.who.int/substance_abuse/publications/global_alcohol_report/gsr_2018/en, accessed 11 June 2020).
[12] Noncommunicable diseases and mental health: About 9 voluntary global targets. Geneva, World Health Organization, 2013 (http://www.who.int/nmh/ncd-tools/definition-targets/en, accessed 11 June 2020).
[13] SAFER: Preventing and reducing alcohol-related harms. Geneva: World Health Organization, 2018 (https://www.who.int/substance_abuse/safer/msb_safer_framework.pdf, accessed 11 June 2020).
[14] Sornpaisarn B, Shield KD, Cohen JE, Schwartz R, Rehm J. The association between taxation increases and changes in alcohol consumption and traffic fatalities in Thailand. J Public Health (Oxf). 2016 Dec 2;38(4):e480-e488. DOI: https://doi.org/10.1093/pubmed/fdv163.
[15] Lai T, Habicht J. Decline in alcohol consumption in Estonia: Combined effects of strengthened alcohol policy and economic downturn. Alcohol Alcohol. 2011 Mar-Apr;46(2):200-203. DOI: https://doi.org/10.1093/alcalc/agr001.
[16] Alcohol policy impact case study. The effects of alcohol control measures on mortality and life expectancy in the Russian Federation. Copenhagen: WHO Regional Office for Europe, 2019 (http://www.euro.who.int/en/health-topics/disease-prevention/alcohol-use/publications/2019/alcohol-policy-impact-case-study-the-effects-of-alcohol-control-measures-on-mortality-and-life-expectancy-in-the-russian-federation-2019, accessed 11 June 2020).

---

[17] Bruni L, Diaz M, Barrionuevo-Rosas L, et al. Global estimates of human papillomavirus vaccination coverage by region and income level: a pooled analysis. Lancet Glob Health. 2016 Jul;4(7):e453-463. DOI: https://doi.org/10.1016/S2214-109X(16)30099-7.

[18] Chang MH. Cancer prevention by vaccination against hepatitis B. Recent Results Cancer Res. 2009;181:85-94. DOI: https://doi.org/10.1007/978-3-540-69297-3_10.

[19] Skyrud KD, Myklebust TÅ, Bray F, et al. How many deaths from colorectal cancer can be prevented by 2030? A scenario-based quantification of risk factor modification, screening, and treatment in Norway. Cancer Epidemiol Biomarkers Prev. 2017 Sep;26(9):1420-1426. DOI: https://doi.org/10.1158/1055-9965.epi-17-0265.

[20] Holme Ø, Løberg M, Kalager M, et al. Effect of flexible sigmoidoscopy screening on colorectal cancer incidence and mortality: a randomized clinical trial. JAMA. 2014 Aug 13;312(6):606-615. DOI: https://doi.org/10.1001/jama.2014.8266.

[21] Allemani C, Matsuda T, Di Carlo V, et al.; CONCORD Working Group. Global surveillance of trends in cancer survival 2000–14 (CONCORD-3): analysis of individual records for 37 513 025 patients diagnosed with one of 18 cancers from 322 population-based registries in 71 countries. Lancet. 2018 Mar 17;391(10125):1023-1075. DOI: https://doi.org/10.1016/s0140-6736(17)33326-3.

[22] Breast cancer: prevention and control. Geneva, World Health Organization, 2013 (http://www.who.int/cancer/detection/breastcancer/en/index3.html, accessed 11 June 2020).

[23] Kenneth KW. Esophageal cancer prevention. Gastroenterol Hepatol (N Y). 2016 Dec;12(12):780-782.

[24] Lip and oral cavity cancer treatment (adult) (PDQ®) – Health professional version. Bethesda: National Institutes of Health, National Cancer Institute, 2019 (https://www.cancer.gov/types/head-and-neck/hp/adult/lip-mouth-treatment-pdq, accessed 11 June 2020).

[25] Stein JP, Lieskovsky G, Cote R, et al. Radical cystectomy in the treatment of invasive bladder cancer: long-term results in 1,054 patients. J Clin Oncol. 2001 Feb 1;19(3):666-675. DOI: https://doi.org/10.1200/jco.2001.19.3.666.

[26] Manoharan M, Ayyathurai R, Soloway MS. Radical cystectomy for urothelial carcinoma of the bladder: an analysis of perioperative and survival outcome. BJU Int. 2009 Nov;104(9):1227-1232. DOI: https://doi.org/10.1111/j.1464-410x.2009.08625.x.

[27] Survival statistics for non-melanoma skin cancer. Toronto, Canadian Cancer Society, 2019 (http://www.cancer.ca/en/cancer-information/cancer-type/skin-non-melanoma/prognosis-and-survival/survival-statistics, accessed 11 June 2020).

[28] Testicular cancer treatment (PDQ®) – Health professional version. Bethesda: National Institutes of Health, National Cancer Institute, 2019 (https://www.cancer.gov/types/testicular/hp/testicular-treatment-pdq, accessed 11 June 2020).

[29] Cancer. Geneva, World Health Organization, 2019 (http://www.who.int/cancer/en, accessed 11 June 2020).
